# Diagnostics of Data-Driven Models: Uncertainty Quantification of PM7 Semi-Empirical Quantum Chemical Method

**DOI:** 10.1038/s41598-018-31677-y

**Published:** 2018-09-05

**Authors:** James Oreluk, Zhenyuan Liu, Arun Hegde, Wenyu Li, Andrew Packard, Michael Frenklach, Dmitry Zubarev

**Affiliations:** 1 Department of Mechanical Engineering, University of California at Berkeley, Berkeley, California 94720-1740 USA; 2 IBM Almaden Research Center, 650 Harry Road, San Jose, California, 95136 USA

## Abstract

We report an evaluation of a semi-empirical quantum chemical method PM7 from the perspective of uncertainty quantification. Specifically, we apply Bound-to-Bound Data Collaboration, an uncertainty quantification framework, to characterize (a) variability of PM7 model parameter values consistent with the uncertainty in the training data and (b) uncertainty propagation from the training data to the model predictions. Experimental heats of formation of a homologous series of linear alkanes are used as the property of interest. The training data are *chemically accurate*, i.e., they have very low uncertainty by the standards of computational chemistry. The analysis does not find evidence of PM7 consistency with the entire data set considered as no single set of parameter values is found that captures the experimental uncertainties of all training data. A set of parameter values for PM7 was able to capture the training data within ±1 kcal/mol, but not to the smaller level of uncertainty in the reported data. Nevertheless, PM7 was found to be consistent for subsets of the training data. In such cases, uncertainty propagation from the chemically accurate training data to the predicted values preserves error within bounds of chemical accuracy if predictions are made for the molecules of comparable size. Otherwise, the error grows linearly with the relative size of the molecules.

## Introduction

Uncertainty quantification (UQ) is a critical diagnostic for the models that are positioned as a source of actionable predictions. Computational models of this kind are abundant in chemistry. One can think of kinetic models^1–3^, classical force-fields^4,5^, semi-empirical Hamiltonians^6–8^, approximate density functionals^9–13^ and the blossoming field of machine learning/deep learning models^14–19^.

One of the challenges of the model selection and curation is their tendency to proliferate uncontrollably if there is flexibility in the model’s form and fitting to reference data is involved. The rich landscape of approximate density functionals^20^ is a case to the point. Models produced within data-centric framework that are application-oriented will inevitably face the same challenge at a much larger scale^21,22^.

Currently, models that require parameter fitting are compared on the basis of some form of the fitting error^23^. Our goal here is to develop a framework for model-data diagnostics, that informs users about the model from the point of view of the UQ. First, given a set of training data with known, presumably low, uncertainty, we want to be able to establish if there is a domain of the model parameters that captures all training data within their uncertainty. Second, knowing the domain of parameter values that are consistent with the training data, we want to establish how the variance in the parameter values propagates into the uncertainty of the model predictions.

There is a noticeable growth of interest in the assessment of uncertainty of the models used in computational chemistry (see e.g., refs^24–27^ and citations therein). A recent analysis of the error assessment in computational chemistry arrives at the following conclusion^28^: “… a procedure for quantifying the uncertainty associated with computational models, in particular with quantum chemical calculations, is mandatory despite their first-principles character. Otherwise, it may be difficult to draw meaningful conclusions. Unfortunately, this procedure is neither well established nor straightforward.” Earlier we demonstrated how applying the Bound-to-Bound Data Collaboration framework^24,29,30^ for UQ can be incorporated into DFT framework in order to quantify and improve uncertainty of the predictions^31^. In the present study, we apply the same framework as a diagnostic tool for semi-empirical quantum chemical model PM7^32^.

Semi-empirical model chemistries are popular because of the low computational overhead that makes it possible to tackle large systems in a relatively short time. Interpretable mechanistic nature of these methods and straight-forward manner in which empirical data can be incorporated are the reasons why semi-empirical quantum chemistry finds a wide range of ubiquitous applications and sees ongoing development^33,34^.

The goal of the presented study is to determine if (a) there is a single set, i.e., feasible set, of PM7 parameter values that satisfies uncertainty bounds of the training data and (b) it is possible to achieve predictions outside the training data with the uncertainty as low as the uncertainty of the training data.

## Methods and Data

### Bound-to-Bound Data Collaboration

This section provides a brief overview of Bound-to-Bound Data Collaboration (B2BDC), with a focus on the methodological details specific to the present study. A more detailed discussion can be found elsewhere^24,29,30,35,36^.

B2BDC is a deterministic framework for uncertainty quantification, where uncertain parameters are constrained by combining models and experimental data. Let {*M*_*e*_(*x*)}_*e* = 1, …, *N*_ denote a collection of models mapping from a common parameter space to various scalar-valued quantities of interest (QOIs). For a given parameter vector *x* ∈ ${\mathbb{R}}$^*n*^, the expression *M*_*e*_(*x*) evaluates the model prediction for *e*-th QOI. Experimental observations of the QOIs come in B2BDC in the form {[*L*_*e*_,*U*_*e*_]}_*e* = 1, …, *N*_, where the uncertainty is characterized by a lower bound, *L*_*e*_ and a upper bound *U*_*e*_ for the *e*-th QOI. Oftentimes, there is prior knowledge to confine the parameters, *x*, to a set ${\mathcal H}$ ⊂ ${\mathbb{R}}$^*n*^. Prior knowledge can be represented as a set of constraints on either individual model parameters, e.g., −1 ≤ *x*_1_ ≤ 1, or can represent relationships between parameters, e.g., 0 ≤ 5*x*_1_ + *x*_2_ ≤ 2. Constrained by both prior knowledge on *x* and experimental uncertainty bounds, each QOI has an associated feasible set of parameters for which model evaluations agree with the corresponding experimental bounds:

1 $${ {\mathcal F} }_{e}=\{x\in  {\mathcal H} :{L}_{e}\le {M}_{e}(x)\le {U}_{e}\},\,\,e=1,2,\ldots ,N.$$

Naturally, one is often interested in the set of parameters which satisfies multiple observations:

2 $${ {\mathcal F} }_{I}={\cap }_{e\in I}{ {\mathcal F} }_{e},$$

where *I* ⊂ {1, 2, …, *N*} is the index set of the QOIs. For example, ${\mathcal F}$_1:3,5_ denotes the feasible set associated with QOIs, *e* = 1, 2, 3, 5. If the set ${\mathcal F}$_1:*N*_ is nonempty, the models and data are said to be *consistent*. Consistency is a form of model validation, ensuring that a model parameter vector exists that evaluates within the experimental uncertainty bounds for all *N* QOIs. When such a parameter vector does not exist the set ${\mathcal F}$_1:*N*_ is empty, the models and data are said to be *inconsistent*. If the models can be accurately represented by polynomial surrogates, this notion of consistency can be efficiently and provably quantified through constrained optimization^35^. A polynomial surrogate is a model which mimics the behavior of an underlying simulation model as close as possible, while being computationally cheaper to evaluate^37^. In cases where the models are not well characterized by simple surrogates but are computationally inexpensive to evaluate, direct sampling can provide a means of assessing consistency. To prove consistency, we must find at least one parameter vector *x* in ${\mathcal F}$_1:*N*_. However, this becomes very challenging when the prior parameter space is large and there are many QOI models whose experimental bounds must be satisfied.

In B2BDC, the prediction for a particular model, say *M*_*p*_(*x*), amounts to establishing bounds on the range of a model prediction, subject to model parameters being within a feasible set, ${\mathcal F}$_*I*_:

3 $$[\mathop{{\rm{\min }}\,}\limits_{x\in { {\mathcal F} }_{I}}{M}_{p}(x),\,\mathop{{\rm{\max }}}\limits_{x\in { {\mathcal F} }_{I}}\,{M}_{p}(x)].$$

The prediction model need not be a member of the collection of models {*M*_*e*_(*x*)}_*e* = 1, …, *N*_. In certain cases, Eq. 3 can be efficiently solved via constrained optimization^30^. Note, inner approximations to the prediction interval can be found by evaluating the prediction model for a set of parameter values in *ℱ*_*I*_. The inner approximation can be interpreted as saying the prediction model can be no more predictive than the computed bounds and a wider prediction interval indicates greater uncertainty in the prediction.

**Figure 1:**
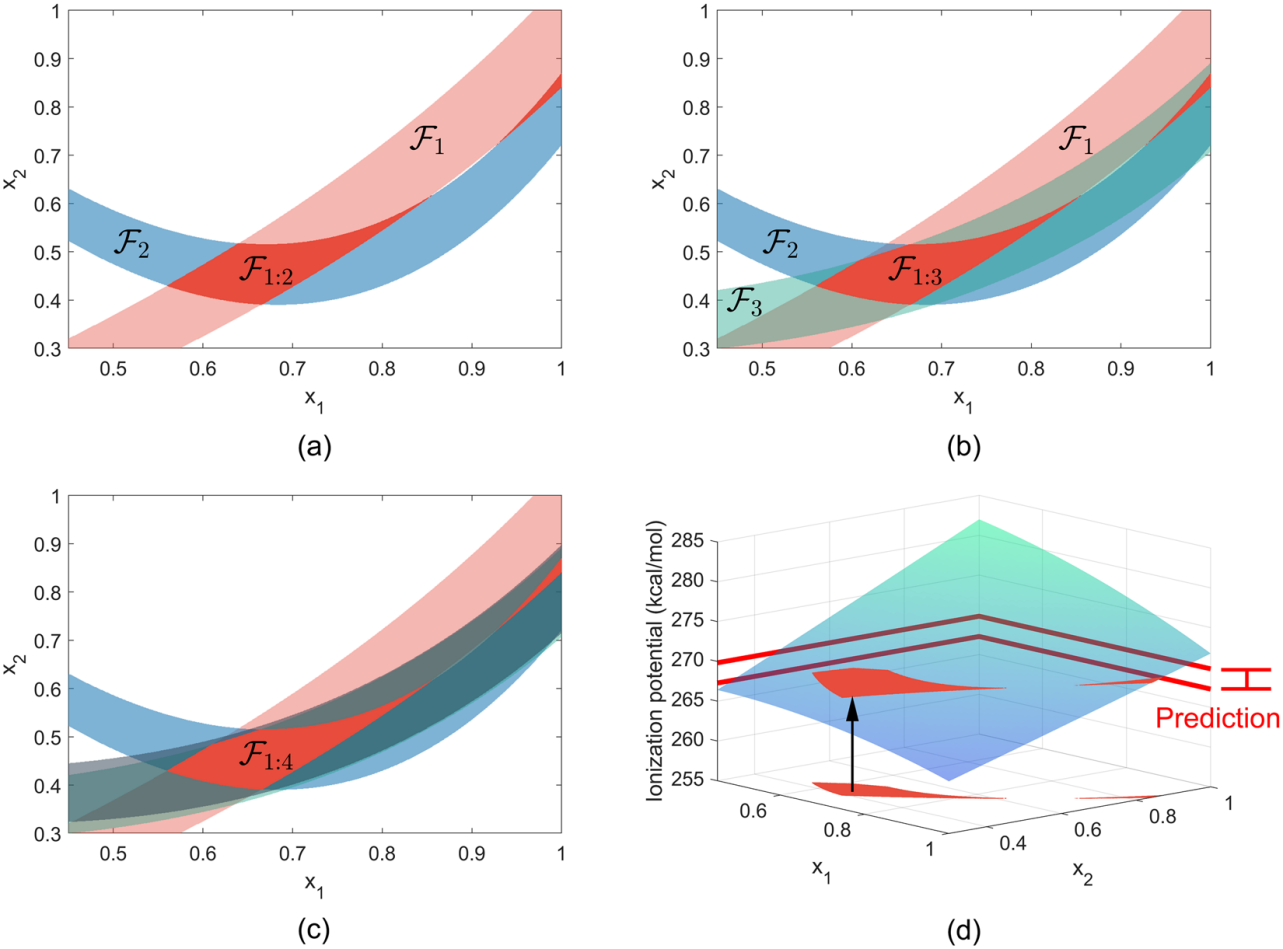
Illustration of the B2BDC methodology, specifically the intersection of feasible sets, for the problem described in Edwards *et al*.^31^. The shaded regions shown in Panels a, b and c are individual feasible set, ${\mathcal F}$_*e*_, which are a set of model parameters that can predict the ionization potential of a particular water cluster within its respective uncertainty. Panel a: individual feasible sets for dimer and trimer configurations of water, ${\mathcal F}$_1_ and ${\mathcal F}$_2_ respectively. The intersection of these two regions is the feasible set ${\mathcal F}$_1:2_, shown in dark red. Panel b: the addition of the feasible set for the tetramer configuration of water, ${\mathcal F}$_3_, shown in teal. The intersection is shown in dark red, ${\mathcal F}$_1:3_. Panel c: the feasible set of pentamer configuration of water, shown in dark grey, primarily overlaps with ${\mathcal F}$_3_. The intersection of all individual feasible sets forms ${\mathcal F}$_1:4_, shown in dark red. Panel d: prediction of the ionization potential of a hexamer water cluster on feasible set ${\mathcal F}$_1:4_. Prediction establishes a range of a prediction model as seen in Eq. 3. The resulting prediction interval for the ionization potential is shown on the right-hand side in red.

As an illustrative example of the B2BDC methodology, pertinent to the present work, we select a case investigated recently^31^. The problem is stated as estimating the ionization potential (IP) of water hexamer and its uncertainty interval, given ionization potentials and their respective uncertainties of water dimer, trimer, tetramer and pentamer. In this problem, there are four QOI models for the training-set of water clusters, consisting of dimer, trimer, tetramer and pentamer. A theoretical model of the ionization potential was constructed based on the double-hybrid form of the Density Functional Theory (DFT). Two model parameters, referred here as *x*_1_ and *x*_2_, encapsulated the model variability over their allowable prior ranges (each 0 to 1). Initial sampling over the parameter space and building surrogate models produced individual feasible regions (Eq. 1), for each of the four training-set water clusters labeled ${\mathcal F}$_1_ through ${\mathcal F}$_4_. The top-left panel of Fig. 1 shows the individual feasible sets of dimer and trimer water clusters, ${\mathcal F}$_1_ and ${\mathcal F}$_2_ respectively and their intersection ${\mathcal F}$_1:2_ shown in dark red. Every point of this red region guarantees predictions of the dimer and trimer IPs within their respective uncertainty bounds. The top-right panel adds the feasible set for tetramer water clusters, ${\mathcal F}$_3_ and the bottom-left panel adds further the feasible set for pentamer water clusters, ${\mathcal F}$_4_. The intersection of all the individual feasible regions produces a feasible region, ${\mathcal F}$_1:4_ which is the feasible set of the B2BDC framework described in Eq. 2; shown in dark red in the bottom-left panel of Fig. 1. Every point of ${\mathcal F}$_1:4_ is a combination of the two model parameters that maps to a value for the property we seek to predict, i.e., the ionization potential of the water hexamer, which is consistent with the ionization potentials of the training data. Collectively, all points in ${\mathcal F}$_1:4_ result in an interval of predicted hexamer IP values, shown in the bottom-right panel of Fig. 1. The predicted uncertainty interval encompasses all the uncertainties of the input data and turned out to be significantly smaller, by about a factor of 2, compared to the high-theory calculations.

### Selection of QOIs and experimental data for PM7

There are a multitude of approaches available for validating a computational model to experimental data. The two most important factors when validating a model is the choice of validation data and the specific experimental feature to validate the model by.

In this study, we will be considering a single homologous series, namely linear alkanes. We chose this homologous series for its simplicity and accumulated knowledge. There are 27 adjustable model parameters in PM7 which are used to parameterize the interactions of carbon and hydrogen atoms. Given the problem choice, we should expect the “best case scenario” from the results.

For a given parameterization, PM7 as a model provides many output responses, e.g., vibrational frequencies, ionization potential, HOMO-LUMO energies, heat of formation, etc. Due to the sheer number of model responses, the availability of accurate experimental data will be used to guide the choice of a quantity of interest. We also keep in mind that the primary output value of PM7 is standard heat of formation. Ruscic *et al*.^38^, employing a thermochemical network approach, reported accurate thermochemical values for a set of molecules, including members of linear alkanes, methane to octane (Table 1). Uncertainties listed in Table 1 represents the reported 95% confidence intervals, in conformity to the accepted standard in thermochemistry^39^. Despite the reported values not being purely experimental, the terms “experimental uncertainty” and “experimental bounds” will be used to describe the data in Table 1. In the present study, heats of formation of linear alkanes will be used as our QOIs. The experimental bounds used for the training data are the the reported heat of formation ± the uncertainty. Table 1 also shows the QOI models used in the study, namely the heat of formation of methane, ethane to octane. For sake of simplicity, each QOI model is indexed by the number of carbon atoms.

**Table 1:** List of the QOI models *M*_*e*_, the associated index *e* (equivalent to the number of carbon atoms in the alkane), the reported heat of formation and uncertainty^38^.

*e*	*M*_*e*_	${{\boldsymbol{\Delta }}}_{{\bf{f}}}{{\bf{H}}}_{{\bf{298}}}^{{\bf{o}}}$ (kcal/mol)	Uncertainty
1	${{\rm{\Delta }}}_{{\rm{f}}}{{\rm{H}}}_{298}^{\circ }({{\rm{CH}}}_{4})$	−17.81	±0.01
2	${{\rm{\Delta }}}_{{\rm{f}}}{{\rm{H}}}_{298}^{\circ }({{\rm{C}}}_{2}{{\rm{H}}}_{6})$	−20.06	±0.03
3	${{\rm{\Delta }}}_{{\rm{f}}}{{\rm{H}}}_{298}^{\circ }({{\rm{C}}}_{3}{{\rm{H}}}_{8})$	−25.10	±0.05
4	${{\rm{\Delta }}}_{{\rm{f}}}{{\rm{H}}}_{298}^{\circ }({{\rm{C}}}_{4}{{\rm{H}}}_{10})$	−30.10	±0.06
5	${{\rm{\Delta }}}_{{\rm{f}}}{{\rm{H}}}_{298}^{\circ }({{\rm{C}}}_{5}{{\rm{H}}}_{12})$	−34.98	±0.07
6	${{\rm{\Delta }}}_{{\rm{f}}}{{\rm{H}}}_{298}^{\circ }({{\rm{C}}}_{6}{{\rm{H}}}_{14})$	−39.90	±0.08
7	${{\rm{\Delta }}}_{{\rm{f}}}{{\rm{H}}}_{298}^{\circ }({{\rm{C}}}_{7}{{\rm{H}}}_{16})$	−44.82	±0.11
8	${{\rm{\Delta }}}_{{\rm{f}}}{{\rm{H}}}_{298}^{\circ }({{\rm{C}}}_{8}{{\rm{H}}}_{18})$	−49.78	±0.16

### Motivation for direct sampling

B2BDC typically employs surrogate models to represent an underlying simulation. The use of surrogates makes tasks like optimization and sensitivity analysis much more efficient due to inexpensive evaluations. When the surrogates are polynomials, consistency and prediction can be addressed efficiently and provably. However, we have found constructing surrogates for PM7 particularly challenging for two reasons, as we will detail below with the ${{\rm{\Delta }}}_{{\rm{f}}}{{\rm{H}}}_{298}^{\circ }({{\rm{C}}}_{4}{{\rm{H}}}_{10})$ serving as our example.

First, we do not have a well-defined prior domain, ${\mathcal H}$, to confine our search for feasible parameters as no bounds are specified for these parameters. To address this, we constructed an initial prior domain ${\mathcal H}$ by centering the domain around the PM7 nominal parameter vector^32^, *x*_nom_, which is listed in Supplementary Table S1. The bounds of the prior domain were constructed by selecting perturbations ±*δ*_*i*_ to the *i*-th parameter with all other parameters remained fixed such that the interval defined by *x*_nom,*i*_ + *δ*_*i*_ produced a 10 kcal/mol change in ${{\rm{\Delta }}}_{{\rm{f}}}{{\rm{H}}}_{298}^{\circ }$. We denote this initial region by ${ {\mathcal H} }_{1}=[{x}_{{\rm{nom}}}\pm \delta ]$, as shown in Supplementary Table S1.

**Figure 2:**
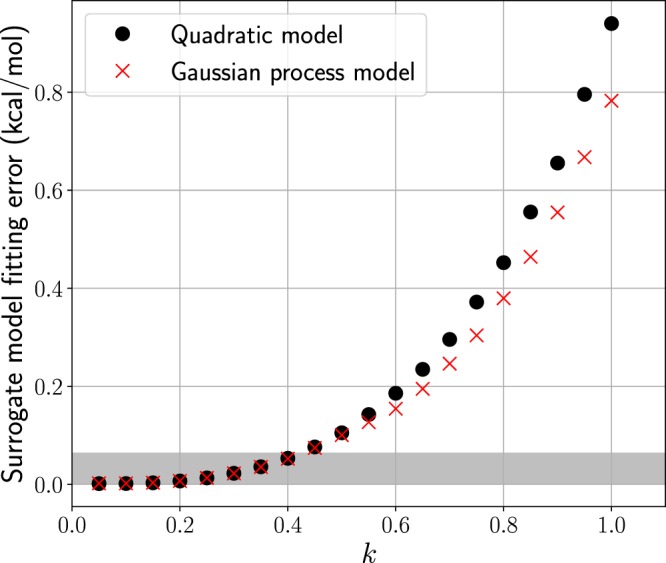
Surrogate model fitting error for the heat of formation of C_4_H_10_ evaluated on a shrinking domain ${ {\mathcal H} }_{k}=[{x}_{{\rm{nom}}}\pm k\times \delta ]$, with *k* ∈ [0, 1]. The nominal PM7 value, *x*_nom_ and *δ* are reported in Supplementary Table S1. As the domain shrinks, by reducing *k*, the surrogate model fitting error, that is the difference between PM7 and the surrogate model, decreases to a point where the error falls below the experimental uncertainty (shown in grey) of 0.06 kcal/mol. Comparison between a quadratic surrogate (black dots) and a Gaussian process model (red crosses) with a constant mean and a squared exponential covariance function is shown. 10-fold cross-validation was used to estimate the fitting error.

Second, our experience has shown that surrogate models are only accurate (to within the experimental uncertainty) on small domains. To illustrate this, we compare the fitting error of a quadratic polynomial and Gaussian process surrogate model on shrinking domains, ${ {\mathcal H} }_{k}=[{x}_{{\rm{nom}}}\pm k\times \delta ]$ where *k* ∈ [0, 1], as shown in Fig. 2. The surrogate model fitting error is the absolute difference between an evaluation of PM7 and an evaluation of the surrogate model over a domain ${\mathcal H}$_*k*_. For each *k*, 7500 Latin Hypercube samples were generated in ${\mathcal H}$_*k*_ to construct both surrogate models. 10-fold cross validation was used to estimate the surrogate model fitting error over each domain. The Gaussian process was implemented by using MATLAB’s *fitrgp* function^40^. As seen in Fig. 2, the Gaussian process deviated by approximately 0.8 kcal/mol from PM7 on the domain ${\mathcal H}$_1_, which is significant given the accuracy of the training data. In order to attain a surrogate model fitting error below our target, the experimental uncertainty, the initial region needed to be reduced by the factor of *k* = 0.4. The reduced domain ${\mathcal H}$_0.4_ is 5.5 × 10^10^ times smaller by volume as compared to the original domain ${\mathcal H}$_1_. Based on this result, we concluded that it would require an intractable number of surrogate models to mimic the behavior of PM7 over the prior region ${\mathcal H}$ within the desired accuracy. Therefore, PM7 will be evaluated directly and forgo any surrogate modeling.

### Direct Sampling for Consistency

In light of the challenges discussed in the preceding section, we approached consistency and prediction by directly sampling ${\mathcal H}$. Still, despite PM7’s fast evaluation time, particularly for fixed geometries, evaluating consistency for multiple QOIs remains a computational challenge. We break this challenge into two tasks: (1) allocating computational resources to sample ${\mathcal H}$; and (2) determining a prior region ${\mathcal H}$ to confine our search for feasible parameters.

#### Computational burden of direct sampling PM7

One of the limitations encountered in the present study was the number of samples we could evaluate for PM7 with the computational resources available. Evaluating PM7 for millions of parameter combinations is an embarrassingly parallel task, but computational efficiency can be improved by reducing an input/output (I/O) bottleneck occurring in the evaluation process.

Throughout the study, the input molecular geometries were obtained via optimization with the PM7 nominal parameter vector, which remained frozen afterwards, during the subsequent direct sampling. The freezing of the molecules geometries was done to avoid any unphysical reorganization or distortion. The consequences of freezing the molecular geometries with the nominal PM7 parameter vector are two-fold. First, using geometry optimization for a given parameter vector can (and usually does) result in different heats of formation as compared to the fixed geometry. Second, parameter vectors which are feasible with respect to the heats of formation, do not guarantee reaching a physically meaningful geometry. All feasible parameter vectors, which would produce unphysical geometries, should ultimately be removed from the feasible set.

The computational chemistry program MOPAC^41^ was used for evaluation of PM7 parameter sets. One of the practical aspects to be solved in working with MOPAC is handling efficiently multiple reading and writing of text files for each execution, which creates a bottleneck in I/O when parallelizing the direct sampling approach. To reduce I/O time, a memory based file system was used, that enabled all files to be read and written into system memory (RAM) instead of a hard disk drive (HDD). For example, using a Seagate 7200 RPM SATA HDD, read and write speeds were 204.0 and 194.9 MB/s, respectively. Using a memory based file system we could read and write at 6020.7 and 5840.6 MB/s, respectively. To relate this to sampling performance, the time to evaluate 10^4^ different parameter values for C_4_H_10_ on a workstation equipped with an Intel Core i7-6700 K processor took 305.0 seconds using the Seagate hard drive and 215.7 using the memory based file system, a speed up of 41%. This speedup in reading and writing MOPAC text files directly translates into more evaluations of PM7 per second.

The MATLAB Parallel Computing Toolbox^40^ was used to write input files, execute PM7 and read output files in parallel. Two workstations, each with an Intel Core-i7 6700 K processor were used to directly sample PM7 and evaluate the heats of formation of a series of alkanes, methane to nonane.

#### Determination of a prior search region

The choice of the prior parameter domain, ${\mathcal H}$, determines all subsequent analysis and hence it needs to be well motivated. Our goal is to assess the predictivity of PM7 for large alkanes while being consistent with experimental data available for small alkanes. Due to limited data, we set aside heptane and octane, the two largest alkanes for which we have experimental bounds, to assess predictivity. Conversely, we also set aside methane and ethane so as not to bias the result. Although the physical properties of methane and ethane may be representative of the homologous series, the chemical properties, i.e., the energetics, are very different as seen through group additivity^42^. Propane and butane were also not included in the initial region search, since a priori it was not known if a parameter vector exist that could simultaneously evaluate the heats of formation of propane, butane, heptane and hexane within the uncertainty in the training data. Based on these considerations, we prioritized our determination of ${\mathcal H}$ on feasible samples for pentane and hexane, ${\mathcal F}$_5:6_.

A non-linear programming solver, *fmincon*^40^, was used to minimize the following objective function, ${\Vert {M}_{\mathrm{5:6}}(x)-{y}_{\mathrm{5:6}}\Vert }_{2}^{2}$, where, *M*_5:6_(*x*) are the heats of formation of pentane and hexane computed by PM7 at the parameter vector *x*. *y*_5:6_ are the reported heats of formation for pentane and hexane from Table 1. The local optimal parameter vector found, *x*_opt_, was feasible for pentane and hexane, therefore ${x}_{{\rm{opt}}}\in { {\mathcal F} }_{\mathrm{5:6}}$. A volume was taken around *x*_opt_ in order to collect samples of ${\mathcal F}$_5:6_. For each sampled point, the heats of formation of pentane and hexane were computed by PM7. 2 × 10^5^ samples were generated by employing a Latin Hypercube design from the volume [*x*_opt_ ± *x*_opt_ × 1 × 10^−3^] and 65 samples were found to be within ${\mathcal F}$_5:6_.

Principal component analysis (PCA) was conducted to identify a rotated coordinate system around the samples of ${\mathcal F}$_5:6_, where all principal components were preserved. Considering the arbitrary choice of the volume used in the sampling of ${\mathcal F}$_5:6_, each PCA direction was extended ten times of that of the ${\mathcal F}$_5:6_ samples. This allowed for an even larger sample region to be considered. A Latin Hypercube design was used to generate uniform samples in the rotated and extended volume.

In total, 5.76 million samples were generated uniformly within the PCA-rotated volume and were used to evaluate PM7 for the heats of formation of nine alkanes, CH_4_ to C_9_H_20_. Shown in Table 2 are the extreme parameter values from the generated samples. There were 164,569 samples which were feasible for at least one alkane in the training set. The number of feasible samples found quickly dropped when considering feasibility with multiple alkanes. For example, there were 19167 samples that were feasible with at least two alkanes, 6193 samples feasible with at least three alkanes, 3110 samples feasible with at least four alkanes, 1989 samples feasible with at least five alkanes and 169 samples feasible with at least six alkanes.

**Table 2:** Extreme parameter values from the search region.

Parameter	min(x)	max(x)
USS_H_	−14.815	−9.5475
BETAS_H_	−10.444	−6.383
ZS_H_	0.85244	1.5053
GSS_H_	10.953	15.822
FN11_H_	0.14962	0.23173
FN21_H_	1.2161	1.4849
FN31_H_	0.83875	1.0458
ALPB_H_	3.9629	4.8467
XFAC_H_	2.3077	2.7852
USS_C_	−52.553	−45.61
UPP_C_	−43.679	−37.301
BETAS_C_	−15.285	−11.804
BETAP_C_	−9.2246	−6.7715
ZS_C_	1.5914	2.2229
ZP_C_	1.501	2.0466
GSS_C_	10.314	13.662
GSP_C_	9.7585	13.092
GPP_C_	9.3945	12.464
GP2_C_	8.6452	11.014
HSP_C_	0.66956	0.77601
FN11_C_	0.045528	0.055036
FN21_C_	4.3632	5.1283
FN31_C_	1.4298	1.7154
ALPB_HC_	0.76974	1.1501
XFAC_HC_	0.15181	0.21488
ALPC_C_	2.3296	3.0615
XFAC_C_	0.71959	0.96953

## Results

### Feasible Set Search

For each of the eight alkanes, a feasible parameter vector could be found that would predict its heat of formation within the experimental bounds. Feasible parameters were also found for all pairwise combinations of alkanes, i.e., a parameter vector could be found that would predict the heat of formation for any two alkanes within their respective experimental bounds. The percentage of feasible samples found for each pair of alkanes in the training data set is reported in Supplementary Table S3. Methane had the smallest percentage of feasible samples for a single alkane and the largest was for octane. This difference in percentages of feasible samples could be due to the uncertainty bounds associated to octane being nearly an order of magnitude larger than that of methane and the difference in molecular structure of methane compared to all others in the homologous series.

From the generated samples, there were 4020 samples of ${\mathcal F}$_5:6_, the alkanes which we had focused our search on. However, from the generated samples, there was no single parameter vector that was able to simultaneously satisfy the uncertainty bounds of all the training data, i.e., ${ {\mathcal F} }_{\mathrm{1:}N}=\varnothing$. Thus the PM7 model and the training data are found to be mutually inconsistent.

The inability to find a parameter vector feasible for all alkanes does not prove the non-existence of such a point. In fact, parameter values that are feasible for all alkanes could lead to potential biases in the prediction because methane and ethane are sufficiently different from the rest of the homologous series with respect to their atomic bonding and hence the energetics. Using parameter values feasible for methane and/or ethane in the prediction of larger alkane properties can result in a prediction interval with smaller uncertainty but in disagreement with experimental data, a problem similar to the bias-variance tradeoff^43^.

Direct sampling the search region led to a fraction of samples being consistent with the experimental data for three alkanes (Supplementary Table S4), 4250 samples were found from ${\mathcal F}$_6:8_. Prediction of all alkanes by ${\mathcal F}$_6:8_ is shown in Supplementary Fig. S2. Feasible sets formed by consecutive alkanes, i.e., adjacent members of the homologous series, where only a few or no samples were found are shown in Supplementary Table S5. The feasible set with the largest number of consecutive alkanes was ${\mathcal F}$_3:8_. Prediction of all alkanes by ${\mathcal F}$_3:8_ is shown in Supplementary Fig. S3. No samples were found from ${\mathcal F}$_2:8_ from direct sampling. Using a genetic algorithm via MATLAB’s *ga* function (and 750 + cpu hours), we were able to obtain a single parameter vector from ${\mathcal F}$_2:8_ (Supplementary Table S2).

For PM7 to predict the heats of formation of eight alkanes to within the uncertainty in the training data turned out to be a challenging task. There exists a set of parameter vectors, specifically 28,696 such sets, each of them simultaneously predicting the heats of formation of all eight alkanes with 95%-confidence chemical accuracy, i.e., within ±1 kcal/mol. Since there is no justification for expanding the uncertainty of the training data, the feasible sets used throughout this study are defined by the reported experimental uncertainties, those reported in Table 1.

**Figure 3:**
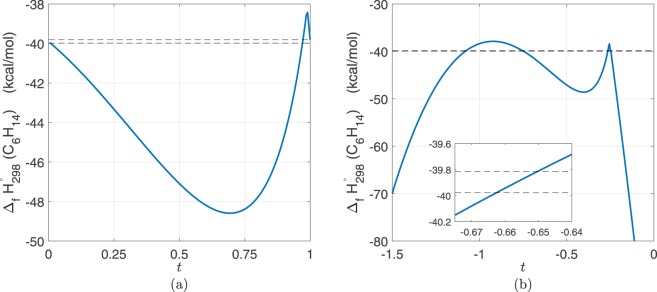
Blue curve is the heat of formation of C_6_H_14_ from PM7 calculations along the line segment defined as *x* = (1 − *t*)*x*_1_ + *tx*_2_, where *x*_1_ and *x*_2_ are two feasible points for C_6_H_14_. The black dashed lines are the respective experimental bounds (see Table 1). Panel a: line segment between two feasible points is not entirely feasible, thus the feasible set of C_6_H_14_ is non-convex. Panel b: extending the line segment beyond *x*_1_, to the left, a new intersection with the experimental bounds is found at *t* = −0.65.

To better understand the shape of a feasible set over the search region, consider two feasible points for C_6_H_14_. The heat of formation of C_6_H_14_ was calculated along the line segment connecting the two feasible points. Figure 3(a) shows that points along the line segment do not remain feasible, thus proving the feasible set is non-convex. Interestingly, if the line segment is extended further, beyond the initial sample region, another intersection with the experimental bounds occurs at *t* = −0.65, shown in Fig. 3(b). This proves that there exist more feasible points for C_6_H_14_ which are outside of the sampled search region.

### Uncertainty of Predictions

**Figure 4:**
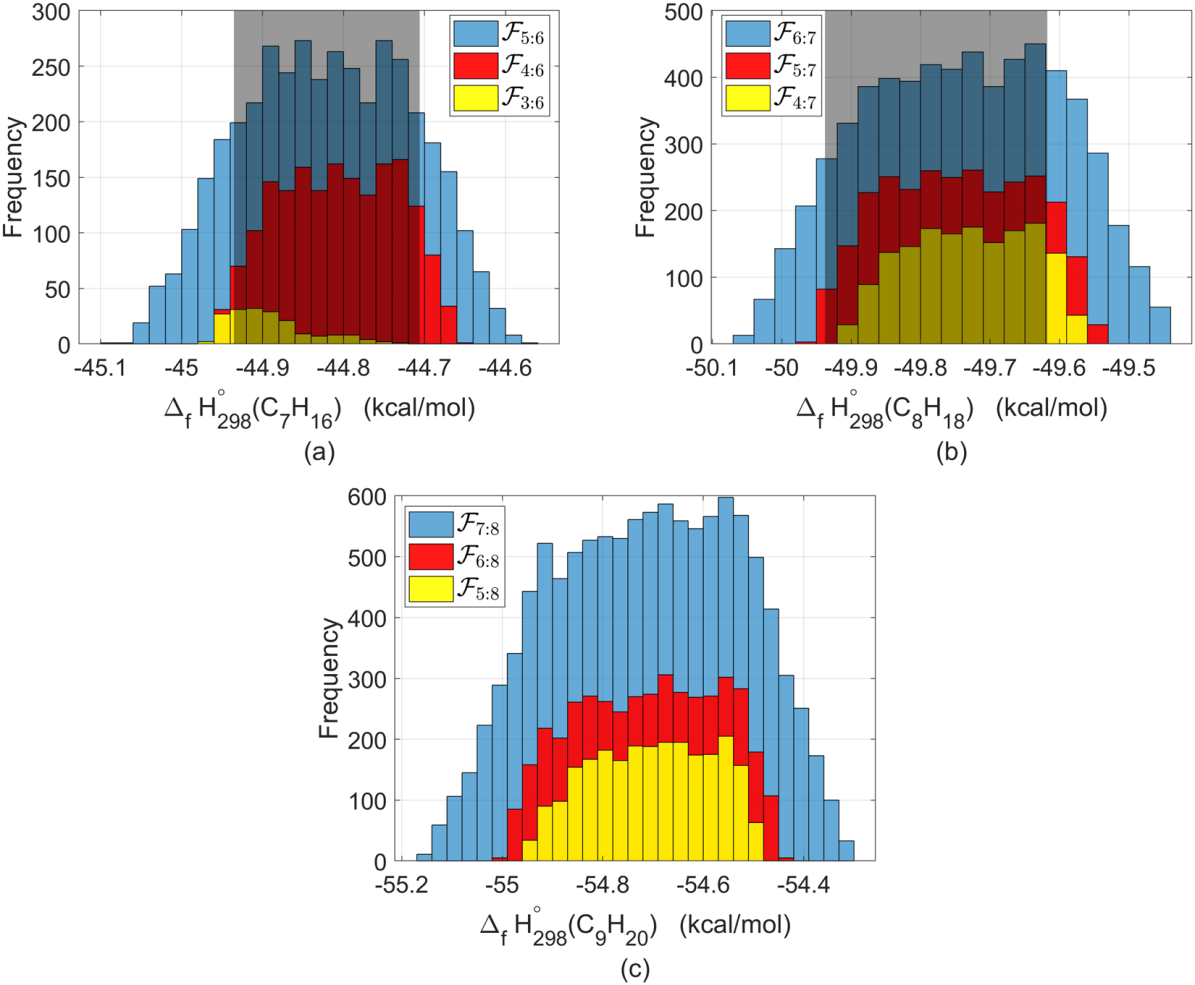
Heat of formation of a larger alkane, predicted from the feasible parameter set of smaller alkanes. The grey shaded region is the experimental interval for the respective alkane from Table 1. Panel a: heat of formation of heptane predicted from samples of ${\mathcal F}$_5:6_, ${\mathcal F}$_4:6_ and ${\mathcal F}$_3:6_. Panel b: heat of formation of octane predicted from samples of ${\mathcal F}$_6:7_, ${\mathcal F}$_5:7_ and ${\mathcal F}$_4:7_. Panel c: heat of formation of nonane predicted from samples of ${\mathcal F}$_7:8_, ${\mathcal F}$_6:8_ and ${\mathcal F}$_5:8_; this case is without experimental data.

In this section, we examine the uncertainty of predictions for larger alkanes using samples consistent with experimental data for smaller alkanes. Prediction of the heat of formation of heptane, octane and nonane were investigated. In the case of heptane and octane, experimental data are available for comparison, but for nonane there are not. In each case, samples consistent with the experimental data for smaller alkanes were used for prediction of the larger alkane forming the histograms shown in Fig. 4. The width of the histograms constitute the prediction uncertainty. The predictions are only inner approximations as the samples were only generated from a portion of the parameter space. Thus, the predicted uncertainty can be no smaller than that which is presented below.

Figure 4(a) depicts the prediction of the heat of formation of heptane. In blue are samples from ${\mathcal F}$_5:6_, i.e, parameter vectors that were feasible for both C_5_H_12_ and C_6_H_14_. The width of the histogram is 0.5 kcal/mol, which is larger than that of the experimental uncertainty of 0.2 kcal/mol (shown in grey), but still within the chemical accuracy (1 kcal/mol in terms of a 95% confidence interval). To further reduce the prediction uncertainty, we can impose additional constraints by considering samples that are also feasible with smaller alkanes. Samples of ${\mathcal F}$_4:6_ and ${\mathcal F}$_3:6_, shown in red and yellow, respectively, are indeed capable of reducing the predicted uncertainty and in the case of ${\mathcal F}$_3:6_ producing a prediction 52.5% smaller than that of ${\mathcal F}$_5:6_.

Following a similar analysis, we examined the prediction for octane, the largest alkane that we had experimental data for comparison. To predict the heat of formation of C_8_H_18_, we considered feasible samples from the preceding two, three and four smaller alkanes, i.e., samples from ${\mathcal F}$_6:7_, ${\mathcal F}$_5:7_ and ${\mathcal F}$_4:7_. The results are shown in Fig. 4(b). The heat of formation of octane predicted by ${\mathcal F}$_6:7_, shown in blue, yielded uncertainty of 0.62 kcal/mol, again within the chemical accuracy. Using samples which were feasible with the preceding three alkanes, ${\mathcal F}$_5:7_, resulted in a prediction uncertainty of 0.42 kcal/mol, a reduction of 31% compared to the prediction by ${\mathcal F}$_6:7_.

The computational results for the alkane without any experimental data for comparison, nonane, are shown in Fig. 4(c). Using samples of ${\mathcal F}$_7:8_, the prediction uncertainty for nonane is 0.85 kcal/mol. We were able to reduce the prediction uncertainty for nonane by 44%, to 0.48 kcal/mol, by considering feasible samples from ${\mathcal F}$_5:8_. Prediction of the heat of formation of nonane using ${\mathcal F}$_3:8_, the feasible set with the largest number of consecutive alkanes found, is given in Supplementary Fig. S1.

**Figure 5:**
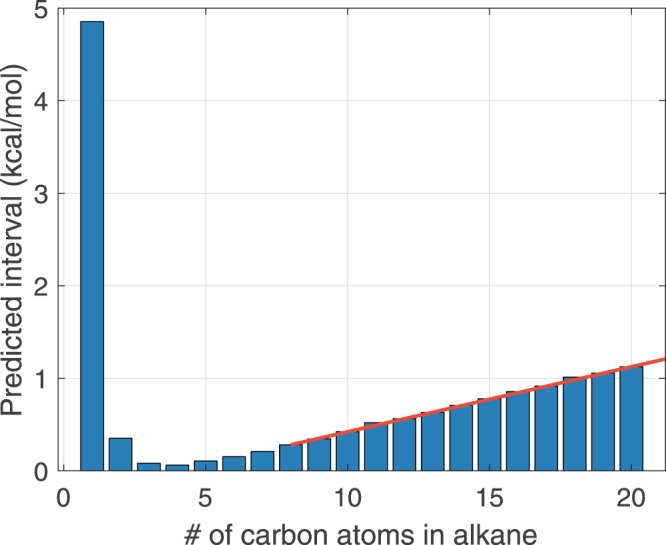
Predicted intervals for alkanes outside of the training data. Prediction was made using samples of ${\mathcal F}$_3:8_. Interval length is defined as the absolute value of the ${{\rm{\max }}}_{x\in { {\mathcal F} }_{\mathrm{3:8}}}{M}_{p}(x)-{{\rm{\min }}}_{x\in { {\mathcal F} }_{\mathrm{3:8}}}{M}_{p}(x)$, where *M*_*p*_(*x*) is the evaluation of PM7 of the *p*-th alkane with the parameter vector, *x*. The red line shown is a linear fit between n-C_8_H_18_ and n-C_20_H_42_.

**Figure 6:**
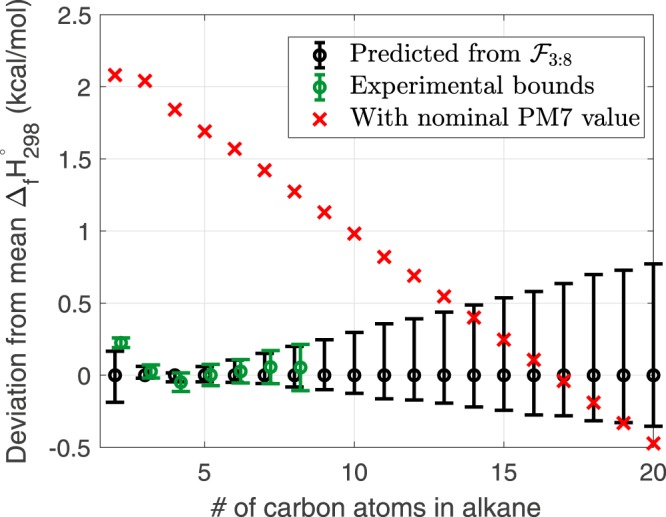
Uncertainty intervals of the heat of formation of ethane to icosane predicted by samples of ${\mathcal F}$_3:8_. The black intervals are prediction by ${\mathcal F}$_3:8_ and with the computed mean positioned at zero and marked with a black circle, the green intervals are the experimental bounds (Table 1) and the red crosses are the heats of formation computed with the PM7 nominal parameter vector.

The predictions for alkanes beyond nonane are shown in Fig. 5. The displayed uncertainty was predicted by using samples of ${\mathcal F}$_3:8_. Two interesting observations can be made from these results. First, using samples from ${\mathcal F}$_3:8_ produces a predicted uncertainty of nearly 5 kcal/mol for methane. This observation is consistent with the fact that methane is sufficiently different from the larger members of the homologous series. Parameter vectors feasible for larger alkanes are not capable of predicting the heat of formation for methane with chemical accuracy. Second, for larger alkanes prediction uncertainty grows linearly with the alkane size. The linearly increasing prediction uncertainty can also be seen in Fig. 6 where C_2_H_6_ through n-C_20_H_42_ are compared against the available experimental data and the nominal PM7 evaluation. Predictions made with samples of ${\mathcal F}$_3:8_ overlap with the experimental bounds from C_3_H_8_ through C_8_H_18_ (by definition of the feasible set ${\mathcal F}$_3:8_), but the nominal parameterization of PM7 deviates by nearly 2 kcal/mol. The PM7 results with the nominal parameter vector intersects with the predicted interval for alkanes C_14_H_30_ through C_19_H_40_.

### Classification of Feasible Points

As was established in Fig. 3, the feasible set of PM7 is non-convex. For a convex feasible set it suffices to map out its boundary in order to determine if any combination of parameters is feasible or not. In the case of PM7, explicitly mapping of the boundary of the feasible set is computationally expensive due to the dimensionality of the problem and the feasible set being non-convex; a viable alternative is to use the data collected via direct sampling and train a binary classifier that distinguishes a “feasible” class of parameter values from the “infeasible” one. As there was no parameter vector found feasible for all alkanes, it is unclear what parameter vectors should be assigned to the “feasible” class. One approach is to only consider parameter values belonging to a specific feasible set, such as ${\mathcal F}$_4:7_, as the “feasible” class. An alternative approach would be to consider the “feasible” class as all parameter vectors that are feasible for at least one alkane. Given the unbalanced nature of the feasible and infeasible classes, i.e., over 5 × 10^6^ samples which are infeasible and only a few thousand samples belonging to any specific feasible set, we choose the latter option and constructed the “feasible” class from all points that are feasible for at least one alkane in the training set.

**Figure 7:**
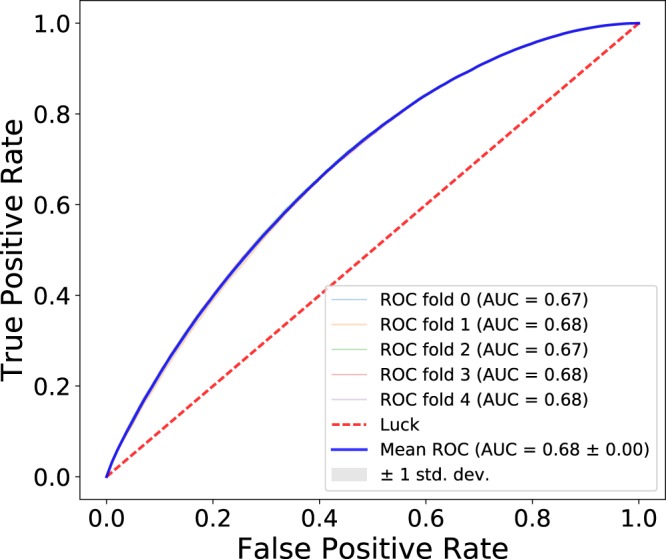
ROC curves and their corresponding AUC for binary classification using a Random Forest model. Classification was between the classes, “feasible” and “infeasible” with the samples obtained via direct sampling. “Feasible” class includes samples that are feasible for at least one alkane. “Infeasible” class includes samples that are not feasible for any alkane. Both classes were down-sampled to 10^5^ samples. Classification performance was assessed by 5-fold cross-validation using 5 × 10^3^ ensemble of random trees.

Binary classification was performed using a Random Forest classifier, as implemented in Scikit-learn library^44^, with equal misclassification costs. A Random Forest classifier is capable of emulating the decision boundary for non-convex and even non-contiguous feasible domains. Both classes were down-sampled to 10^5^ to handle the class imbalance. Down-sampling was performed by using a randomly drawn 10^5^ samples from each class. Performance of the classifier is evaluated in a 5-fold cross-validation via construction of the Receiver Operating Curve (ROC) and computation of the Area Under Curve (AUC) (see Fig. 7). The ROC allows one to gauge the performance of a binary classifier by comparing the true positive and false positive rate across all possible decision thresholds. The true positive rate is the number of feasible samples correctly identified as the “feasible” class over the total number of feasible samples. The false positive rate is the number of samples incorrectly identified as the “feasible” class over the total number of infeasible samples. A perfect classifier would have an ROC curve at the top-left corner of Fig. 7, with a true positive rate of 1 and a false positive rate of 0. For this to occur, all feasible samples would be correctly identified as part of the “feasible” class without misclassifying any infeasible samples as the “feasible” class.

One figure of merit for binary classification is the area under the ROC curve (AUC). The AUC varies between 0 and 1 where a perfect classifier has an AUC of 1 and randomly guessing (luck) would have an AUC of 0.5. With an AUC value of 0.68, we found that there is indeed a non-random structure in the feasible set recovered via direct sampling. Therefore, it is possible to train a classifier that fulfills the same function as the boundary to a feasible set. Of course, for this assessment to be practical, a better performance metric of the classifier should be achieved. One route toward this goal is to collect a sample in the parameter space that conveys a better representation of the spatial extents of the non-convex feasible set found via sampling on a Latin hyper-cube. The inset of Fig. 3(b) depicts a region where a line segment passes through the experimental bounds. This region (and all other intersections) is a 27 dimensional space. Samples in these regions, near the experimental bounds, can help improve the characterization of the feasible set for classification.

## Conclusion

Uncertainty quantification of semi-empirical quantum chemical model PM7 was performed using the B2BDC framework. The obtained results did not show evidence of model consistency even in the “best case” scenario, when we considered heats of formation of a small family of linear alkanes. This finding, however, requires a cautious interpretation.

Lack of evidence of the model consistency with experimental data may imply that either the experimental data has some bias or the model has a deficiency. Given the quality of the experimental data, we tend to think the model inconsistency resides with the parameterization of PM7. To ultimately resolve this question, would require a significantly more engaged investigation of PM7 parameter space whose dimensionality exceeds 27 for organic molecules that include hetero-atoms. The practical lesson of the study is that we could not find a parameter vector feasible for all the training data having searched a finite volume and consumed a certain amount of the computational resources and time. The cost of establishing model consistency is, in itself, a diagnostic that can be used to compare various data-centric models and make an informed selection among them.

Another practical lesson is the source of the encountered lack of consistency. The “problematic” QOIs found were the shortest alkanes, methane and ethane, whose chemical properties are known to be different from the rest of the homologous series. The value of PM7 comes from the low computational cost to evaluate larger molecules. In our study, the heats of formation of larger molecules were found to be consistency with the experimental data. The reported results demonstrate B2BDC’s capability for a deeper analysis into PM7 by evaluating the limits of model consistency in a data-centric setting.

One clear challenge in this study was the inability to accurately represent a significant volume of the parameter space with a surrogate model. The encountered difficulties motivated the development of a tool-set for uncertainty quantification of models with high-dimensional parameter spaces that can have non-convex and even non-contiguous feasible sets. Incorporation of machine learning techniques with the B2BDC framework was shown to be a viable strategy to efficiently tackle cases of this type. Our basic attempt to learn the geometric structure of the identified feasible sets showed encouraging results. Possibility of the accurate binary classification of feasible/infeasible classes suggests a route to sampling strategies that are more efficient than brute-force direct sampling and could rely on a semi-supervised model to search for feasible points.

An equally important aspect of our study concerns uncertainty propagation from the training data that have sub-chemical accuracy to PM7 predictions. We observed that predictions remained within bounds of chemical accuracy for the molecules whose size is close to the size of the molecules in the training set. As expected, improvement of the quality of the feasible sets, i.e., attaining consistency with more alkanes, led to the reduction of the prediction uncertainty. Growth of the prediction error with the difference in the size between the test and training molecules agrees with the size-extensive nature of the selected QOI. This result reinforces intuition that there is a price to switching from interpolation to extrapolation regime. It motivates a large-scale uncertainty quantification study of semi-empirical quantum chemical models where both the size and chemical diversity of the dataset are significantly expanded. In order to facilitate such an effort we are including the parameter vectors and current feasibility labels in the Supplementary Information.

## Electronic supplementary material

Supplementary Information

Feasible Points - Labels 1

Feasible Points - Labels 2

Feasible Points - Parameter Vectors 1

Feasible Points - Parameter Vectors 2

## References

[1] Miller JA, Kee RJ, Westbrook CK (1990). Chemical kinetics and combustion modeling. Annu. Rev. Phys. Chem..

[2] Battin-Leclerc F (2011). Towards cleaner combustion engines through groundbreaking detailed chemical kinetic models. Chem. Soc. Rev..

[3] Frenklach M (2007). Transforming data into knowledge–process informatics for combustion chemistry. Proc. Combust. Inst..

[4] Lemkul JA, Huang J, Roux B, MacKerell AD (2016). An empirical polarizable force field based on the classical drude oscillator model: Development history and recent applications. Chem. Rev..

[5] Warshel A, Kato M, Pisliakov AV (2007). Polarizable force fields: History, test cases and prospects. J. Chem. Theory Comput..

[6] Christensen AS, Kubař T, Cui Q, Elstner M (2016). Semiempirical quantum mechanical methods for noncovalent interactions for chemical and biochemical applications. Chem. Rev..

[7] Yilmazer ND, Korth M (2013). Comparison of molecular mechanics, semi-empirical quantum mechanical and density functional theory methods for scoring protein-ligand interactions. J. Phys. Chem. B.

[8] Thiel W (2013). Semiempirical quantum-chemical methods. WIREs Comput. Mol. Sci..

[9] Burke K (2012). Perspective on density functional theory. J. Chem. Phys..

[10] Wiitala KW, Hoye TR, Cramer CJ (2006). Hybrid density functional methods empirically optimized for the computation of ^13^C and ^1^H chemical shifts in chloroform solution. J. Chem. Theory Comput..

[11] Karton A, Tarnopolsky A, Lamère J-F, Schatz GC, Martin JML (2008). Highly accurate first-principles benchmark data sets for the parametrization and validation of density functional and other approximate methods. derivation of a robust, generally applicable, double-hybrid functional for thermochemistry and thermochemical kinetics. J. Phys. Chem. A.

[12] Zhao Y, Schultz NE, Truhlar DG (2006). Design of density functionals by combining the method of constraint satisfaction with parametrization for thermochemistry, thermochemical kinetics and noncovalent interactions. J. Chem. Theory Comput..

[13] Cui Q, Elstner M (2014). Density functional tight binding: values of semi-empirical methods in an ab initio era. Phys. Chem. Chem. Phys..

[14] Xue LC, Dobbs D, Bonvin AM, Honavar V (2015). Computational prediction of protein interfaces: A review of data driven methods. FEBS letters.

[15] Lusci A, Pollastri G, Baldi P (2013). Deep architectures and deep learning in chemoinformatics: The prediction of aqueous solubility for drug-like molecules. J. Chem. Inf. Model..

[16] Schütt KT, Arbabzadah F, Chmiela S, Müller KR, Tkatchenko A (2017). Quantum-chemical insights from deep tensor neural networks. Nat. Commun..

[17] Hegde G, Bowen RC (2017). Machine-learned approximations to density functional theory hamiltonians. Sci. Rep..

[18] Goh GB, Hodas NO, Vishnu A (2017). Deep learning for computational chemistry. J. Comput. Chem..

[19] McGibbon RT (2017). Improving the accuracy of møller-plesset perturbation theory with neural networks. J. Chem. Phys..

[20] Medvedev MG, Bushmarinov IS, Sun J, Perdew JP, Lyssenko KA (2017). Density functional theory is straying from the path toward the exact functional. Sci..

[21] Cherkasov A (2014). Qsar modeling: Where have you been? where are you going to?. J. Med. Chem..

[22] Mansouri K, Grulke CM, Richard AM, Judson RS, Williams AJ (2016). An automated curation procedure for addressing chemical errors and inconsistencies in public datasets used in qsar modelling. SAR and QSAR Environ. Res..

[23] Peverati R, Truhlar DG (2014). Quest for a universal density functional: the accuracy of density functionals across a broad spectrum of databases in chemistry and physics. Phil. Trans. R. Soc. A.

[24] Russi T, Packard A, Frenklach M (2010). Uncertainty quantification: Making predictions of complex reaction systems reliable. Chem. Phys. Lett..

[25] Faver JC, Yang W, Merz KM (2012). The effects of computational modeling errors on the estimation of statistical mechanical variables. J. Chem. Theory Comput..

[26] Yang X (2018). Atomic radius and charge parameter uncertainty in biomolecular solvation energy calculations. J. Chem. Theory Comput..

[27] Simm, G. N. & Reiher, M. Error-controlled exploration of chemical reaction networks with gaussian processes. *arXiv preprint* available at, https://arxiv.org/abs/1805.09886 (2018). 10.1021/acs.jctc.8b00504 30179500

[28] Simm GN, Proppe J, Reiher M (2017). Error assessment of computational models in chemistry. Chimia.

[29] Frenklach, M., Packard, A. & Seiler, P. Prediction uncertainty from models and data. In *Proceedings of the American Control Conference*, vol. 5, 4135–4140 (IEEE, 2002).

[30] Seiler P, Frenklach M, Packard A, Feeley R (2006). Numerical approaches for collaborative data processing. Optim. Eng..

[31] Edwards DE, Zubarev DY, Packard A, Lester WA, Frenklach M (2014). Interval prediction of molecular properties in parametrized quantum chemistry. Phys. Rev. Lett..

[32] Stewart JJ (2013). Optimization of parameters for semiempirical methods VI: more modifications to the NDDO approximations and re-optimization of parameters. J. Mol. Model..

[33] Dral PO (2016). Semiempirical quantum-chemical orthogonalization-corrected methods: Theory, implementation and parameters. J. Chem. Theory Comput..

[34] Dral PO, Wu X, Spörkel L, Koslowski A, Thiel W (2016). Semiempirical quantum-chemical orthogonalization-corrected methods: Benchmarks for ground-state properties. J. Chem. Theory Comput..

[35] Feeley R, Seiler P, Packard A, Frenklach M (2004). Consistency of a reaction dataset. J. Phys. Chem. A.

[36] Frenklach M, Packard A, Garcia-Donato G, Paulo R, Sacks J (2016). Comparison of statistical and deterministic frameworks of uncertainty quantification. SIAM/ASA J. Uncertainty Quantif..

[37] Box, G. E. & Draper, N. R. *Empirical model-building and response surfaces*. (John Wiley & Sons, 1987).

[38] Ruscic, B. & Bross, D. Active thermochemical tables (ATcT) values based on ver. 1.122 of the thermochemical network, 2016. *avaliable at ATcT.anl.gov* (2017).

[39] Ruscic B (2014). Uncertainty quantification in thermochemistry, benchmarking electronic structure computations and active thermochemical tables. Int. J. Quantum Chem..

[40] MATLAB, statistics and machine learning toolbox, parallel computing toolbox and optimization toolbox release 2017b, The MathWorks Inc. *Natick, MA* (2002).

[41] Stewart, J. J. MOPAC2016. Stewart Computational Chemistry, Colorado Springs, CO. *available at*, http://openmopac.net (2016).

[42] Benson SW (1969). Additivity rules for the estimation of thermochemical properties. Chem. Rev..

[43] Hastie, T., Tibshirani, R. & Friedman, J. *The Elements of Statistical Learning, vol. 2* (Springer Series in Statistics, 2009).

[44] Pedregosa F (2011). Scikit-learn: Machine learning in Python. J. Machine Learning Research.

