# Supplementary material for: Diagnostics of Data-Driven Models: Uncertainty Quantification of PM7 Semi-Empirical Quantum Chemical Method
Source: Sci Rep. 2018 Sep 5;8:13248. doi: 10.1038/s41598-018-31677-y (PMC6125339; doi:10.1038/s41598-018-31677-y)
Supplement: Supplementary file 1 — Supplementary Information [file 41598_2018_31677_MOESM1_ESM.pdf]

Supplementary Information - Diagnostics of Data-Driven Models: Uncertainty Quantification of PM7 Semi-Empirical Quantum Chemical Method

James Oreluk*, Zhenyuan Liu*, Arun Hegde*, Wenyu Li*, Andrew Packard*, Michael Frenklach†, Dmitry Zubarev‡

Feasible parameter vectors and feasibility labels are provided in two sets of CSV files. The feasibility labels files, `feasiblePoints_labels_1.csv` and `feasiblePoints_labels_2.csv` are of size nSamples-by-8, where column 1 is the feasibility label associated with methane, column 2 is the feasibility label associated with ethane, and so forth to column 8 which is feasibility label for octane. True indicates feasible and false indicates infeasible.

The associated feasible parameters vector files, `feasiblePoints_parameters_1.csv` and `feasiblePoints_parameters_2.csv` are of size nSamples-by-27, where each row is a parameter vector. The columns are the 27 parameters listed in Table S1. The feasibility of a parameter vector is found in the corresponding row of the associated feasibility labels file.

*Department of Mechanical Engineering, University of California at Berkeley, Berkeley, California 94720-1740

†Corresponding author: frenklach@berkeley.edu, Department of Mechanical Engineering, University of California at Berkeley, Berkeley, California 94720-1740

‡IBM Almaden Research Center, 650 Harry Road, San Jose, California 95136

Table S1: PM7 nominal parameter vector and the δ associated with a 10 kcal/mol change in the heat of formation of C_4H_{10}, where each parameter value was perturbed, one-at-a-time from its nominal value.

**Parameter**	**Value**	$\boldsymbol{\delta}$
$\mathbf{USS_H}$	-11.07	0.29033
$\mathbf{BETAS_H}$	-8.3897	0.069276
$\mathbf{ZS_H}$	1.2602	0.017434
$\mathbf{GSS_H}$	14.15	0.36839
$\mathbf{FN11_H}$	0.17785	0.009959
$\mathbf{FN21_H}$	1.4287	0.33716
$\mathbf{FN31_H}$	0.99132	0.077376
$\mathbf{ALPB_H}$	4.0512	0.54877
$\mathbf{XFAC_H}$	2.8456	4.875
$\mathbf{USS_C}$	-51.373	0.11427
$\mathbf{UPP_C}$	-40.135	0.084482
$\mathbf{BETAS_C}$	-14.415	0.12922
$\mathbf{BETAP_C}$	-7.8937	0.067632
$\mathbf{ZS_C}$	1.9422	0.012886
$\mathbf{ZP_C}$	1.7087	0.007471
$\mathbf{GSS_C}$	12.347	0.13671
$\mathbf{GSP_C}$	11.933	0.18749
$\mathbf{GPP_C}$	10.452	0.8254
$\mathbf{GP2_C}$	9.3855	0.031369
$\mathbf{HSP_C}$	0.80263	0.11697
$\mathbf{FN11_C}$	0.045888	0.005604
$\mathbf{FN21_C}$	5.0371	1.5601
$\mathbf{FN31_C}$	1.5887	0.12723
$\mathbf{ALPB_{HC}}$	1.0387	0.007624
$\mathbf{XFAC_{HC}}$	0.20458	0.002002
$\mathbf{ALPC_C}$	2.6557	0.024061
$\mathbf{XFAC_C}$	0.93782	0.034605

Table S2: Parameter vector of $\mathcal{F}_{2:8}$ found via global optimization with a genetic algorithm using MATLAB's *ga* function.

Parameter	Value
$\mathbf{USS_H}$	-14.577
$\mathbf{BETAS_H}$	-7.3947
$\mathbf{ZS_H}$	1.1558
$\mathbf{GSS_H}$	15.096
$\mathbf{FN11_H}$	0.21973
$\mathbf{FN21_H}$	1.3555
$\mathbf{FN31_H}$	0.89818
$\mathbf{ALPB_H}$	4.0908
$\mathbf{XFAC_H}$	2.6264
$\mathbf{USS_C}$	-50.896
$\mathbf{UPP_C}$	-42.679
$\mathbf{BETAS_C}$	-12.204
$\mathbf{BETAP_C}$	-8.0123
$\mathbf{ZS_C}$	1.9807
$\mathbf{ZP_C}$	1.5976
$\mathbf{GSS_C}$	12.653
$\mathbf{GSP_C}$	10.104
$\mathbf{GPP_C}$	9.8942
$\mathbf{GP2_C}$	9.8562
$\mathbf{HSP_C}$	0.77597
$\mathbf{FN11_C}$	0.051751
$\mathbf{FN21_C}$	4.8582
$\mathbf{FN31_C}$	1.6624
$\mathbf{ALPB_{HC}}$	1.0878
$\mathbf{XFAC_{HC}}$	0.17728
$\mathbf{ALPC_C}$	2.5316
$\mathbf{XFAC_C}$	0.75887

Table S3: Percentage of feasible points found (out of 5.76 million samples) for each alkane and pair of alkanes.

	CH_4	C_2H_6	C_3H_8	C_4H_{10}	C_5H_{12}	C_6H_{14}	C_7H_{16}	C_8H_{18}
CH_4	0.2647	0.0036	0.0028	0.0027	0.0026	0.0019	0.0025	0.0030
C_2H_6		0.4253	0.0148	0.0113	0.0093	0.0077	0.0088	0.0100
C_3H_8			0.4063	0.0318	0.018	0.0133	0.0140	0.0159
C_4H_{10}				0.4441	0.0572	0.0317	0.0297	0.0307
C_5H_{12}					0.4172	0.0698	0.0501	0.0461
C_6H_{14}						0.3869	0.1070	0.0750
C_7H_{16}							0.4723	0.1915
C_8H_{18}								0.5723

Table S4: Largest percentage of feasible samples for three alkanes, indexed by QOI model.

$\mathcal{F}_{i,j,k}$	**Percentage of feasible samples**
6, 7, 8	0.0738
5, 6, 7	0.0488
5, 6, 8	0.0434
5, 7, 8	0.0432
4, 5, 6	0.0312
4, 5, 7	0.0285
4, 6, 7	0.0282
4, 5, 8	0.0278
4, 7, 8	0.0266
4, 6, 8	0.0260

Table S5: Feasible sets of consecutive alkanes which had a few or no feasible samples, indexed by the QOI model.

**QOI Index**	**Number of feasible samples**
1, 2, 3	7
1, 2, 3, 4	4
1, 2, 3, 4, 5	0
2, 3, 4, 5	7
5, 6, 7	2809
5, 6, 7, 8	2431
4, 5, 6, 7, 8	1431
3, 4, 5, 6, 7, 8	145

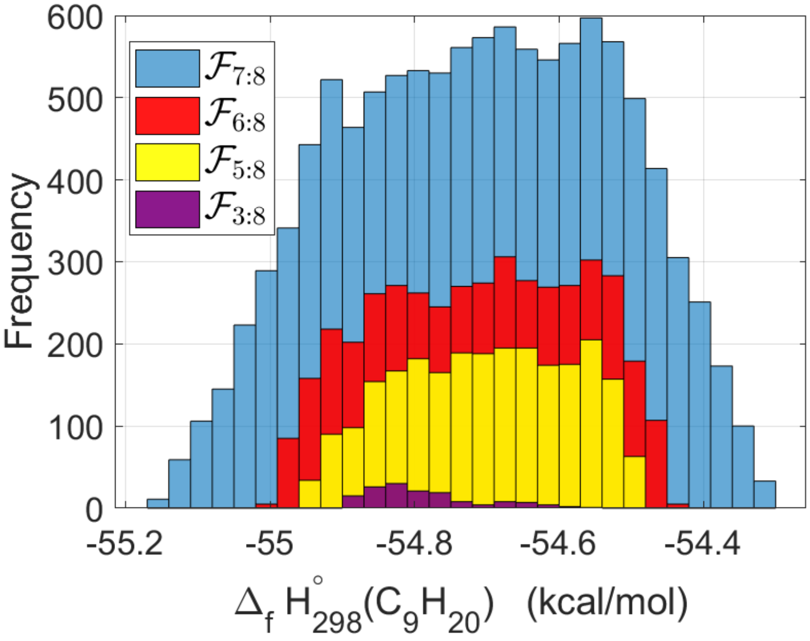

Figure S1: Predicted heat of formation of nonane by feasible samples of smaller alkanes. Using samples of $\mathcal{F}_{3:8}$ reduce the uncertainty in the predicted heat of formation by 40.5% compared to the prediction from samples of $\mathcal{F}_{7:8}$.

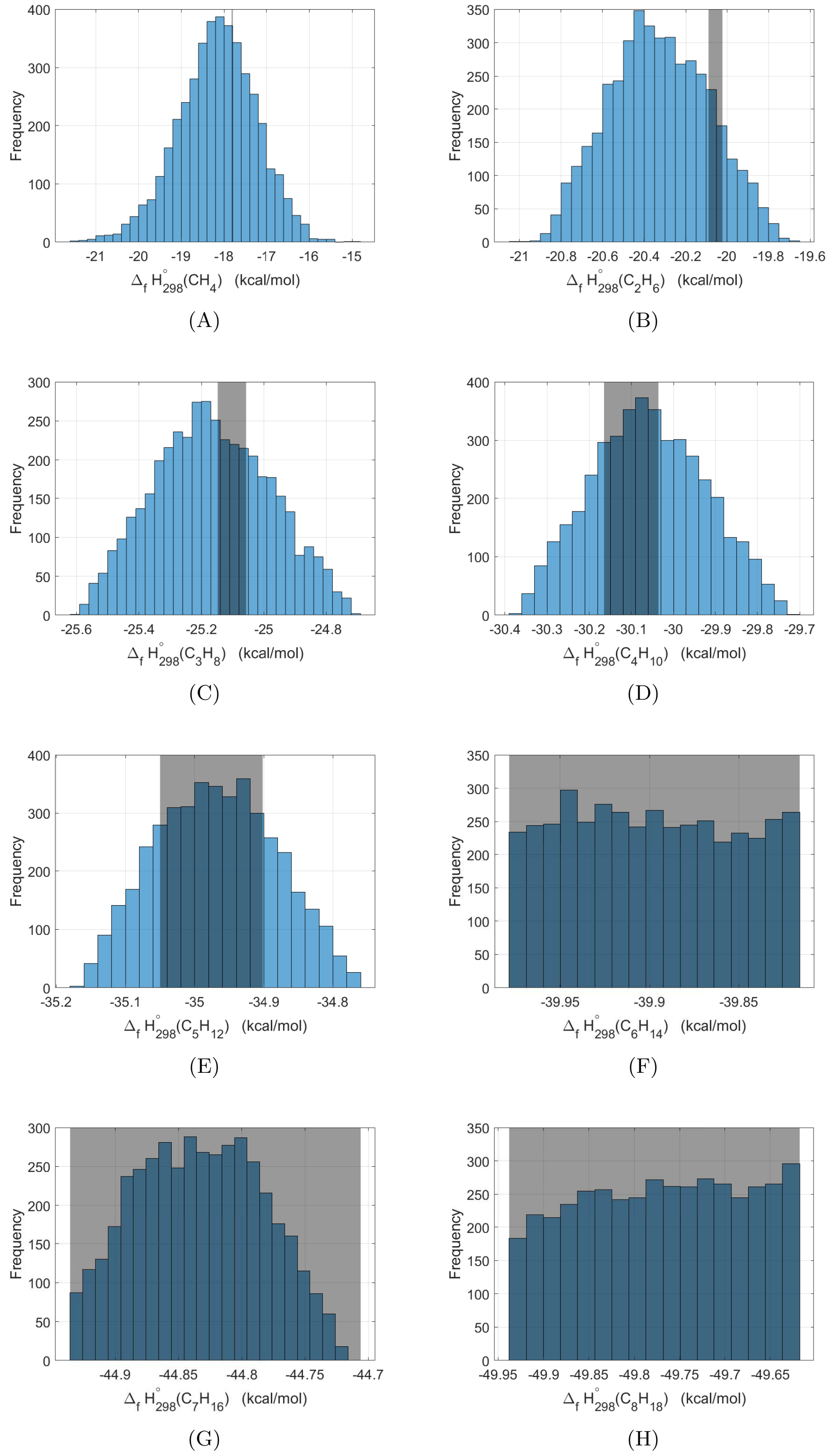

Figure S2: Predicted heat of formation of methane, ethane, to octane from samples of $\mathcal{F}_{6:8}$. The grey shaded region is the respective alkanes experimental interval shown in Table 1.

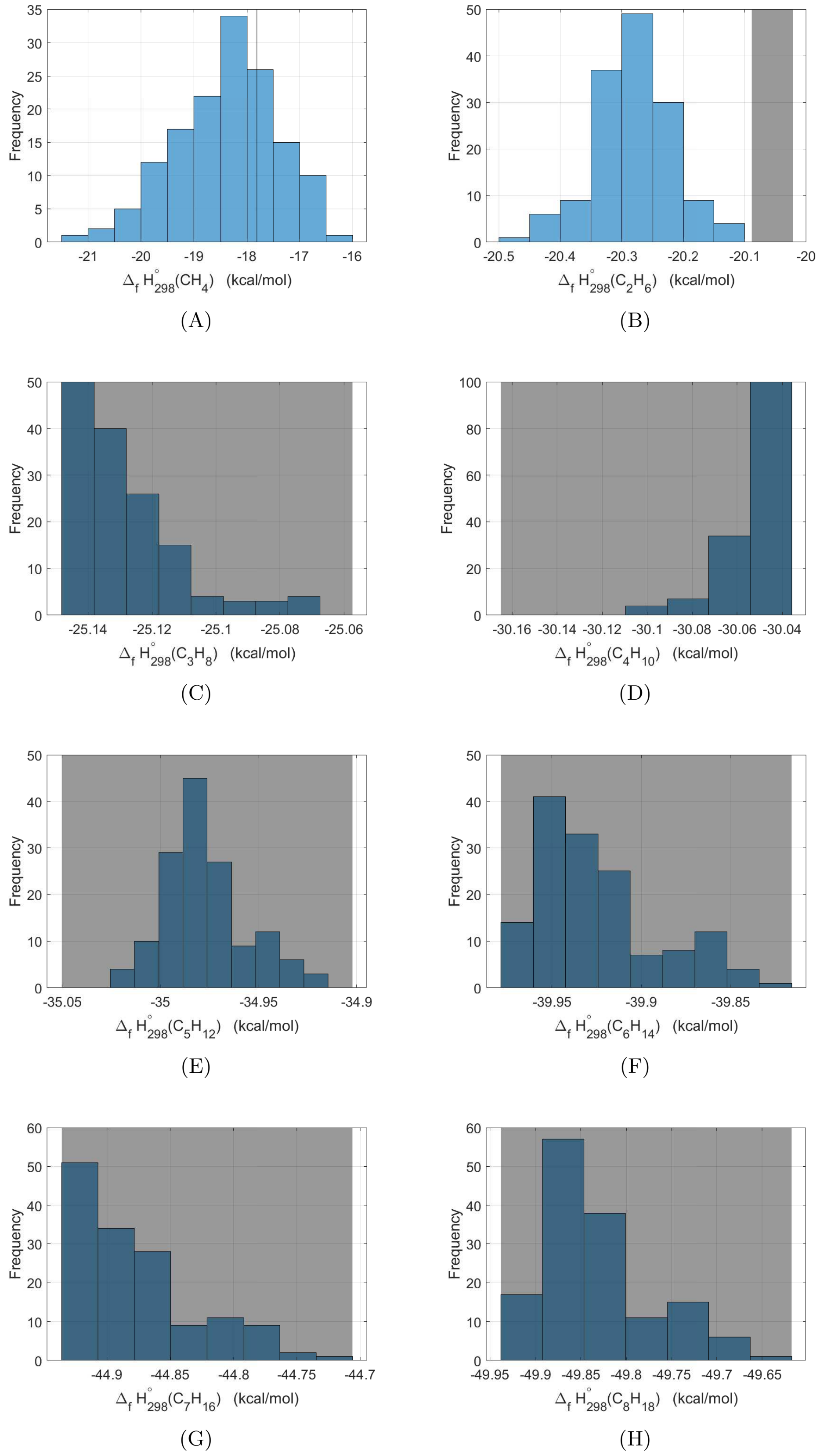

Figure S3: Predicted heat of formation of methane, ethane to octane from samples of $\mathcal{F}_{3:8}$. The grey shaded region is the respective alkanes experimental interval shown in Table 1.

Acknowledgments

Work at UCB was supported by the Department of Energy, National Nuclear Security Administration, under Award Number(s) DE-NA0002375. The views and opinions of authors expressed herein do not necessarily state or reflect those of the United States Government or any agency thereof.
